# Partial Annuloplasty Rings in the Repair of Functional Ischemic Mitral Regurgitation

**DOI:** 10.7759/cureus.8419

**Published:** 2020-06-03

**Authors:** Muhammad Aqeel Kunwar, Imran Muhammad, Muhammad N Khan, Begum Sumreen, Najia A Soomro, Khalid Naseeb, Ali Imran

**Affiliations:** 1 Cardiac Surgery, National Heart Institute, Kuala Lumpur, MYS; 2 Institute of Medical Technology, Dow University of Health Sciences, Karachi, PAK; 3 Interventional Cardiology, National Institute of Cardiovascular Diseases, Karachi, PAK; 4 Stem Cell Research Laboratory, Sindh Institute of Urology and Transplantation, Karachi, PAK; 5 Cardiology, Liaquat National Hospital, Karachi, PAK; 6 Cardiology, National Institute of Cardiovascular Diseases, Karachi, PAK; 7 Research and Statistics, Tabba Institute of Heart Disease, Karachi, PAK

**Keywords:** annuloplasty, mitral regurgitation, acute myocardial infarction

## Abstract

Background

Acute myocardial infarction (MI) is the leading cause of worldwide cardiac morbidities and mortalities. Mitral regurgitation (MR) is a common complication of MI. The severity of ischemic MR (IMR) can range widely, both clinically and hemodynamically. Mitral valve (MV) repair by lifting annuloplasty is a surgical procedure used to correct the pathology of IMR. The immediate outcomes of this technique have not yet been determined. The present study, therefore, evaluated the immediate results of MV annuloplasty performed to complement MV repair in patients with IMR.

Methodology

All adult patients with IMR who underwent lifting posterior mitral annuloplasty (LPMA) plus concomitant coronary artery bypass grafting (CABG) were included. Immediate outcomes were evaluated by transesophageal color Doppler echocardiography. The frequency of successful outcomes was compared in patients with different baseline characteristics.

Results

Posterior mitral annuloplasty was successful in 93.1% of patients, including in 92.8% of men and 94.1% of women. The percentages of successful immediate outcomes differed significantly in patients with and without diabetes and hypertension, and in patients with two- and three-vessel disease.

Conclusion

LPMA resulted in a high percentage of successful immediate outcomes in patients with IMR. Further studies should compare rates of immediate, intermediate, and late outcomes of this technique.

## Introduction

Acute myocardial infarction (MI) is the most common and life-threatening type of cardiac emergency. Mitral regurgitation (MR) is an important sequelae of ischemia that can be seen during and after the acute phase of MI, being observed in up to 25% of patients [1]. In addition, MR is the most common type of heart valve disease in the United States, affecting approximately 1.7% of the US population [2].

Risk factors predicting poorer long-term cardiovascular mortality after acute MI include advanced age, anterior MI, expansion of the infarction, and recurrent ischemia [3]. Transient ischemic MR (IMR) is common during early phase acute MI, rarely leading to hemodynamic compromise [4]. However, the involvement of multiple chordae tendineae or papillary muscle ruptures can result in an acute overload of atrial and left ventricular (LV) volumes, often leading to marked hemodynamic deterioration and frequently presenting as cardiogenic shock [5]. The severity of IMR can range widely, from clinically silent to clinically and hemodynamically obvious, and may be detected only as an incidental finding during cardiac catheterization or Doppler echocardiography [6]. The widespread availability, ease of use, and non-invasive nature of Doppler echocardiography have made it the standard tool for diagnosing MR as an excessive load on the LV, leading to various compensatory steps, including the regulation of the myocardium and blood circulation [7].

Mitral valve (MV) repair is a surgical procedure that has become the most attractive approach to the treatment of severe MR, with excellent immediate and long-term results [8]. One method of MV repair, remodeling annular annuloplasty, which was designed to restore the shape of the distorted fibrous ring of the MV, has yielded predictable and stable results [9]. Ring annuloplasty has allowed the development of additional methods to respond to various emerging lesions [10]. Surgical correction of the posterior prolapse (PL) can be performed using a technique called the "French correction," involving quadrilateral resection, the transfer of normal cords to another area of prolapse and remodeling of the annuloplasty [11]. Classification of this physiopathological triad of severe MR has enhanced the ability to choose the optimal surgical repair technique for any MV pathology [12,13]. This classification included clear descriptions of the etiology of MR as degenerative or ischemic; of valve damage as cord rupture or perforation; and of valve dysfunction [14]. Methods of MV reconstructive surgery specific to these three classes of valve dysfunction have been developed, including for specific types of lesions and pathologies [15].

Mitral annuloplasty is an essential component of most MV repair methods for MR regurgitation. The circular geometry of the MV has been studied to maintain the annular movement and improve repair results after annuloplasty [16]. However, most traditional semi-rigid, rigid, and flexible rings were attached to the valve ring on a flat plane, resulting in limited circular movement during the cardiac cycle. Although some flexible rings have been manufactured to provide natural annular movement, both the transverse and flexible dimensions of the valve become almost stationary, because the entire flexible ring is attached to the MV (doctoral dissertation: Jin C. Annular Flattening in Mitral Valve Prolapse. The Chinese. University of Hong Kong). For double-layer winding, reducing the size of the transverse valve by annuloplasty may be unnecessary and may interfere with leaflet motion. Reducing the cross-section of the septal-lateral dimension, however, may be important, as the main factors facilitating MV repair are a reduction in transverse annular size and an increase in the angle of the mitral hinge. The shape of the annuloplasty ring is also important, creating annular non-planarity and curvature of the valve to reduce valve stress and increase repair longevity [17]. A new type of mitral annuloplasty band was developed to lift the medial part of the posterior ring on the base of both commissural ends, eliminating the anterior leaflet and the commissures [18].

This study evaluated the immediate results of innovative MV annuloplasty, which was performed to complement MV repair in patients with IMR.

## Materials and methods

This descriptive cross-sectional study was designed to evaluate the immediate outcome after combined coronary artery bypass grafting (CABG) and MV repair by posterior lifting annuloplasty among patients treated at the Tabba Heart Institute, Karachi, Pakistan. The study subjects included 335 male and female patients with ischemic heart disease, aged <50-70 years, who underwent lifting posterior mitral annuloplasty (LPMA) for MV repair. Patients with ST-segment elevated and non-ST-segment elevated MI with grade 3+ ischemic MV regurgitation, diagnosed on color Doppler echocardiography, were included in this study. Patients with coexisting valvular heart disease other than mitral, cardiogenic shock, stroke, renal dialysis, or chronic obstructive pulmonary disease were excluded. This study was approved by the College of Physicians and Surgeons of Pakistan and the institutional ethical review board committee, and informed consent was obtained taken all patients and their blood relatives.

Patient characteristics were recorded on pre-designed forms. Data recorded included patient demographic characteristics (e.g., name, age, and gender), hospital registration number, type of coronary vessel disease (two- or three-vessels), the presence of co-morbidities (diabetes and hypertension), type of MI (ST-segment elevated or non-ST-segment elevated), post-procedure color Doppler echocardiography grade, and final outcome (i.e., successful or unsuccessful resolution of MR grade). Cardiopulmonary bypass was performed using a median sternotomy approach, involving standard ascending aortic arterial and direct vena cava cannulation under moderate hypothermia. Following infusion of cold blood cardioplegic solution to achieve cardiac arrest, the MV was exposed through a left atriotomy in the Sondergaard groove. We followed the process as described by Song et al. [4]. After repair, CABG was completed, and cardiopulmonary bypass terminated in a standard manner. Immediately after the procedure, transesophageal color Doppler echocardiography was performed to determine the resolution of IMR grade, with an MR grade of 0/1+/2+ considered successful.

Collected data were entered and analyzed using IBM Statistical Package for the Social Sciences for Windows, Version 21.0 (IBM Corp., Armonk, NY). The cutoff p-value for statistical significance was ≤ 0.05.

## Results

Table 1 shows the demographic and clinical characteristics of the 335 patients with IMR. The 335 patients included 250 men (74.6%) and 85 women (25.4%), of mean age 58.70 ± 7.258 years. Of these patients, 148 (44.2%) were aged 51-60 years and 134 (40.0%) were aged 61-70 years, whereas only 53 (15.8%) were aged <50 years. Two hundred patients (59.7%) had diabetes, including 62 (72.9%) of the women and 138 (55.2%) of the men (p = 0.003). Similarly, 259 patients (77.3%) had hypertension, including 79 (92.9%) women and 180 (72.0%) men (p <0.001). Most of these patients (n=282; 84.2%), had three-vessel disease, which was more frequent in men (86.4%) than in women (77.6%); whereas only 53 (15.8%) had two-vessel disease, which was more frequent in women (22.4%) than in men (13.6%). Of the 335 patients, 110 (32.8%) had ST-segment elevated MI, and 225 (67.2%) had non-ST-segment elevated MI.

**Table 1:** Demographic and clinical characteristics of patients with ischemic mitral regurgitation

Characteristic variables	Male	Female	P-value	Total
N	250	85		335
Age (years)	58.72±7.500	58.61±6.641	0.903	58.70±7.282
Age groups				
<50	41 (16.4%)	12 (14.1%)	0.165	53 (15.8%)
51-60	103 (41.2%)	45 (52.9%)		148 (44.2%)
61-70	106 (42.4%)	28 (32.9%)		134 (40.0%)
Comorbid conditions				
Diabetes	138 (55.2%)	62 (72.9%)	0.003	200 (59.7%)
Hypertension	180 (72.0%)	79 (92.9%)	< 0.001	259 (77.3%)
Types of coronary vessel disease				
Two-vessel disease	34 (13.6%)	19 (22.4%)	0.044	53 (13.6%)
Three-vessel disease	216 (86.4%)	66 (77.6%)		282 (84.2%)
Type of myocardial infarction				
ST-segment elevated	89 (35.6%)	21 (24.7%)	0.065	110 (32.8%)
Non-ST-segment elevated	161 (64.4%)	64 (75.3%)		225 (67.2%)

**Figure 1:**
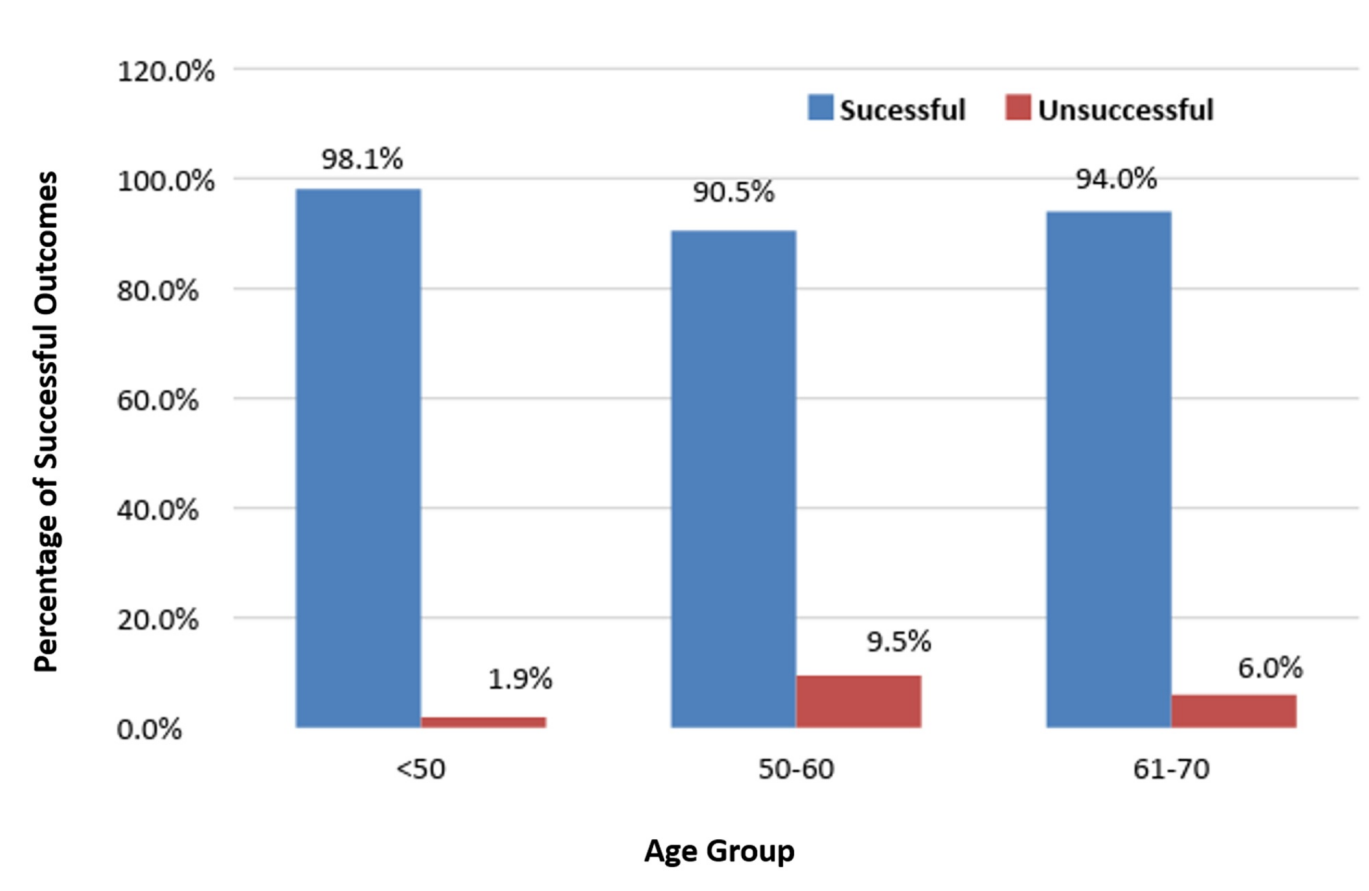
Immediate outcomes among patients in different age groups

Evaluation of immediate outcomes showed that lifting annuloplasty was successful in 312 (93.1%) patients and unsuccessful in only 23 (6.9%) in Table 2. Success rates were similar in men (92.8%) and women (94.1%), but slightly higher in patients with two-vessel (98.1%) than three-vessel disease (92.2%) and in patients with ST-segment elevated (96.4%) than non-ST-segment elevated (91.6%). Success rates were similar in patients with and without diabetes and in patients with and without hypertension. Assessments of postoperative outcomes among the different age groups showed that treatment was highly successful in patients aged < 50 years (98.1%) and 61-70 years, but less successful in patients aged 50-60 years (Figure 1).

**Table 2:** Immediate outcomes of lifting posterior mitral annuloplasty according to different categories of the patients with ischemic mitral regurgitation

	Successful	Unsuccessful	P-value	Total
Male	232 (92.8%)	18 (7.2%)	0.678	250 (74.6%)
Female	80 (94.1%)	5 (5.9%)		85 (25.4%)
Two-vessel disease	52 (98.1%)	1 (1.9% )	0.146	53 (15.8%)
Three-vessel disease	260 (92.2%)	22 (7.8%)		282(84.2%)
ST-segment elevated	106 (96.4%)	4 (3.6%)	0.102	110 (32.8%)
Non-ST-segment elevated	206 (91.6%)	19 (8.4%)		225 (67.2%)
Type 2 diabetic	187(93.5%)	13 (6.5%)	0.747	200 (59.7%)
Non-diabetic	125 (92.6%)	10 (7.4%)		135 (40.3%)
Hypertensive	239 (92.3%)	20 (7.7%)	0.256	59 (77.3%)
Non-hypertensive	73 (96.1%)	3 (3.9%)		76 (22.7%)
Total	312 (93.1%)	23 (6.9%)	NA	335(100%)

## Discussion

The present study reports immediate outcomes of partial annuloplasty among a series of patients with IMR. Functional IMR, which occurs in up to 20% of patients after MI, is usually mild or moderate in severity but is associated with an increased mortality rate [19]. Surgical MV repair has become the method of choice for the correction of MR [20]. Devices used to date for the repair of ischemic MVs include posterior annuloplasty bands (flexible or rigid) and complete or partial rings [21]. To date, however, no large trials have compared the relative efficacy of these devices. The success rates of saddle shape annuloplasty of the MV have been quantified using various imaging modalities, with optimal valve performance shown to be dependent on band shape [22]. These findings led to the introduction of a new generation of saddle-shaped annuloplasty rings, which were found to be superior to flat rings in maintaining more physiologic patterns of annular and leaflet geometry [14]. We hypothesized that, despite the negative influence of undersized annuloplasty rings on leaflet cooptation, undersized saddle-shaped annuloplasty rings for IMR would have a substantial beneficial influence on leaflet cooptation.

The combination of CABG and MV annuloplasty repair was successful in 93.1% of patients with moderate to severe IMR, as shown by transesophageal color Doppler echocardiography. The midterm evaluation showed high success rates for LPMA to enhance leaflet coaptation in MV repair [4]. LPMA using an innovative annuloplasty strip in MV repair, resulted in rare relevant complications, with low rates of recurrent regurgitation and valve-related reoperation. The 30-day mortality rate was 0.9%, and nine late deaths (2.6%) occurred. The mean overall survival rate at five years was 96.0%±1.1%.

MV annuloplasty, in conjunction with CABG, is regarded as the optimal treatment for severe functional IMR, with a high success rate [23]. Repair limited to a Duran flexible annuloplasty ring resulted in a success rate of 98%, as shown by intraoperative transesophageal color Doppler echocardiography, similar to our findings [24]. Furthermore, CABG plus mitral annuloplasty with an asymmetric ring resulted in a high survival rate, including a hospital death rate of 10.0%, with one patient undergoing immediate reoperation for residual grade ≥2 MR and a reoperation success rate of 97.6% [25]. Heart failure symptoms in patients with IMR were also improved by asymmetric annuloplasty, which restored the geometry and competence of the mitral apparatus, provided good mid-term outcomes, and helped LV reverse remodeling. This technique resulted in a 3.7% operative mortality rate in patients with moderate IMR [26]. Other studies have reported mortality rates of 4%-6% [27]. Moreover, the use of saddle-shaped annuloplasty rings for MV repair improved leaflet cooptation in patients with IMR [28].

The primary limitations of the present study included the inability to compare these results with those of other studies because few studies have assessed the immediate outcome of partial annuloplasty in patients with IMR. Geometric changes in the MV apparatus were not assessed by three-dimensional echocardiography, only by two-dimensional echocardiography. Also, all patients received the same model of annuloplasty ring, preventing comparisons between annuloplasty rings of different designs. Furthermore, this study did not assess long-term outcomes.

## Conclusions

Patients requiring surgical revascularization for suspected ischemic MR should be carefully assessed by preoperative echocardiography to determine the severity of the MR. The combination of mitral annuloplasty and CABG in patients with moderate to severe ischemic MR may improve functional capacity, LV reverse remodeling, and MR severity. CABG plus reduction annuloplasty reduces MR and relieves symptoms in patients with functional IMR. Further studies are needed to compare the immediate, intermediate, and late outcomes of partial annuloplasty in patients with MR caused by different etiologies. Although long-term prognosis remains guarded, this single-center study shows the intermediate-term benefits of such an approach.
